# TRACER: twin-contrastive learning for fine-grained and rare spatial domain identification in spatial transcriptomics

**DOI:** 10.1093/bioinformatics/btag805

**Published:** 2026-10-30

**Authors:** Qiaoming Liu, Xin Lu, Bingsen Song, Qianran Wei, Wenzhe Feng, Kexin Guo, Jian Wang, Murong Zhou, Yuming Zhao, Guohua Wang

**Affiliations:** School of Artificial Intelligence, Henan University, Zhengzhou 450000, China; Faculty of Computing, Harbin Institute of Technology, Harbin 150001, China; Zhengzhou Advanced Research Institute, Harbin Institute of Technology, Zhengzhou 450000, China; School of Artificial Intelligence, Henan University, Zhengzhou 450000, China; School of Computer Science and Artificial Intelligence, Northeast Forestry University, Harbin 150040, China; School of Artificial Intelligence, Henan University, Zhengzhou 450000, China; School of Artificial Intelligence, Henan University, Zhengzhou 450000, China; School of Computer and Information Engineering, Henan University of Economics and Law, Zhengzhou 450000, China; School of Computer Science and Artificial Intelligence, Northeast Forestry University, Harbin 150040, China; School of Computer Science and Artificial Intelligence, Northeast Forestry University, Harbin 150040, China; Faculty of Computing, Harbin Institute of Technology, Harbin 150001, China; Zhengzhou Advanced Research Institute, Harbin Institute of Technology, Zhengzhou 450000, China

## Abstract

**Motivation:**

Fine-grained and rare spatial domains are difficult to identify in spatial transcriptomics because they contain few spots, show subtle transcriptional differences, and are strongly influenced by neighboring regions.

**Results:**

We present TRACER, a twin-contrastive learning framework that integrates a graph convolutional autoencoder with neighborhood-aware instance-level and cluster-level contrastive learning. By jointly preserving local spatial continuity and enhancing domain separability, TRACER achieved competitive and generally robust performance compared with representative spatial domain identification methods across multiple spatially resolved transcriptomic datasets, including human dorsolateral prefrontal cortex generated by 10× Visium, mouse brain datasets generated by STARmap, osmFISH, and BARISTAseq, and human breast cancer. TRACER recovered major cortical and anatomical structures, improved the identification of rare or transitional domains in the mouse somatosensory cortex, and revealed candidate functionally distinct small domains within a broadly annotated breast cancer region.

**Availability and implementation:**

The source code of TRACER is available at https://github.com/luxin-heart/TRACER.

## 1 Introduction

Spatial transcriptomics has enabled gene expression to be measured within its native tissue context, providing a powerful approach for dissecting tissue architecture and local microenvironmental heterogeneity. These data reveal that tissues are not only organized into large anatomical compartments, but also contain narrow layers, transition zones, boundary regions, and small microenvironmental niches that may occupy only a limited number of measured spots (Cervilla *et al.* 2026). As spatial transcriptomics is increasingly applied to complex organs and heterogeneous disease tissues, computational methods are needed to identify major tissues and subtle local structures from noisy, high-dimensional spatial expression profiles (Yuan *et al.* 2024).

Spatial domain identification is a central task in spatial transcriptomics analysis. It aims to partition a tissue section into spatially coherent regions with distinct molecular profiles (Hou *et al.* 2024, Lei *et al.* 2024), thereby supporting tissue annotation (Lu *et al.* 2025), spatially variable gene detection (Chen Xuanwei *et al.* 2025, Li Zhijian *et al.* 2025), cell–cell interaction analysis (Yan *et al.* 2025), trajectory inference (Li Zhongzhan *et al.* 2025), and microenvironment characterization (Lin *et al.* 2026). Early analyses often relied on general-purpose clustering methods, such as K-means, Louvain, Leiden, and Gaussian mixture clustering, which primarily rely on gene expression similarity and do not explicitly model spatial organization (Yuan *et al.* 2024). To better incorporate spatial context, methods that use statistics and spatial priors, such as BayesSpace (Zhao *et al.* 2021), Giotto (Dries *et al.* 2021), and stLearn (Pham *et al.* 2023), have introduced neighborhood information, histological context, and spatially aware clustering strategies. Recently, graph-based deep learning techniques, including SpaGCN (Hu *et al.* 2021), STAGATE (Dong and Zhang 2022), and SEDR (Xu *et al.* 2024), have emerged. These methods leverage graph convolution, graph attention, or graph autoencoding to learn embeddings that are informed by spatial data. Additionally, self-supervised and contrastive learning frameworks, such as GraphST (Long *et al.* 2023), stDCL (Yu *et al.* 2025), and STAIG (Yang *et al.* 2025), have further enhanced spatial representation learning by leveraging augmented views, graph structures, and histological images. These methods have substantially advanced the identification of major spatial domains across diverse datasets.

Fine-grained domains (Lee *et al.* 2025, Yousefi *et al.* 2026) refer to small spatial substructures that exist within, between, or along broader tissue regions. In contrast, rare domains (Swain *et al.* 2024, Fa *et al.* 2026) are defined as spatially coherent areas with low spot abundance but anatomical or functional significance (Kleshchevnikov *et al.* 2022). These domains pose challenges because they contain few spots, exhibit weak or gradual transcriptional differences, and are heavily influenced by surrounding regions (Li *et al.* 2024). Current methods often do not optimize for this scenario: spatial smoothing can combine small domains with adjacent larger regions, graph-based aggregation may not ensure local continuity, and contrastive or self-supervised learning objectives typically focus on global representation consistency rather than preserving low-abundance domains. Thus, the key challenge is to learn representations that preserve neighborhood-level continuity while maintaining cluster-level separability, allowing subtle domains to be resolved without being over-smoothed or fragmented (Xu *et al.* 2024).

To tackle this challenge, we developed TRACER, a twin-contrastive learning framework for fine-grained spatial domain identification, with a focus on rare-domain-related and transitional-layer settings in spatial transcriptomics. TRACER employs a graph convolutional autoencoder to learn representations of spatial spots from gene expression and their spatial neighborhoods and further incorporates two complementary contrastive objectives. The neighborhood-aware instance-level contrastive module considers spatially adjacent spots as context-aware positive samples, allowing local tissue neighborhoods to guide the representation learning process directly. Meanwhile, the cluster-level contrastive module aligns cluster assignment structures across augmented views, promoting clear domain organization. An entropy-based regularization is employed to mitigate issues with degenerate clustering. Together, these modules are designed to preserve continuity at the neighborhood level while maintaining separability at the cluster level. This design aims to improve the identification of subtle spatial domains and transitional tissue structures while reducing the risk of over-smoothing or fragmentation.

Moreover, we evaluated TRACER on human and mouse brain datasets and human breast cancer tissue. TRACER accurately recovered major cortical layers and complex anatomical structures, showed competitive performance in rare-domain-related analyses and superior performance in a cortical transitional-layer subset of the mouse somatosensory cortex, and identified small spatial domains with distinct functional programs within a broadly annotated breast cancer region. These results suggest that TRACER provides an effective framework for identifying both major spatial domains and fine-scale tissue substructures, offering a useful approach for dissecting local spatial heterogeneity in complex tissues.

## 2 Materials and methods

### 2.1 Overview of TRACER

TRACER is a twin-contrastive learning framework for spatial domain identification in spatial transcriptomics, with a focus on fine-grained and low-abundance domains. As illustrated in Fig. 1, TRACER randomly shuffles node feature vectors while preserving the graph topology (Fig. 1A), thereby introducing feature-level perturbation without disrupting the spatial neighborhood structure. TRACER consists of a graph convolutional autoencoder backbone (Fig. 1B) and two complementary contrastive learning modules (Fig. 1C and D). After training, the reconstructed expression profiles are used for fine-grained or rare spatial domain identification, followed by biological downstream tasks (Fig. 1E).

**Figure 1:**
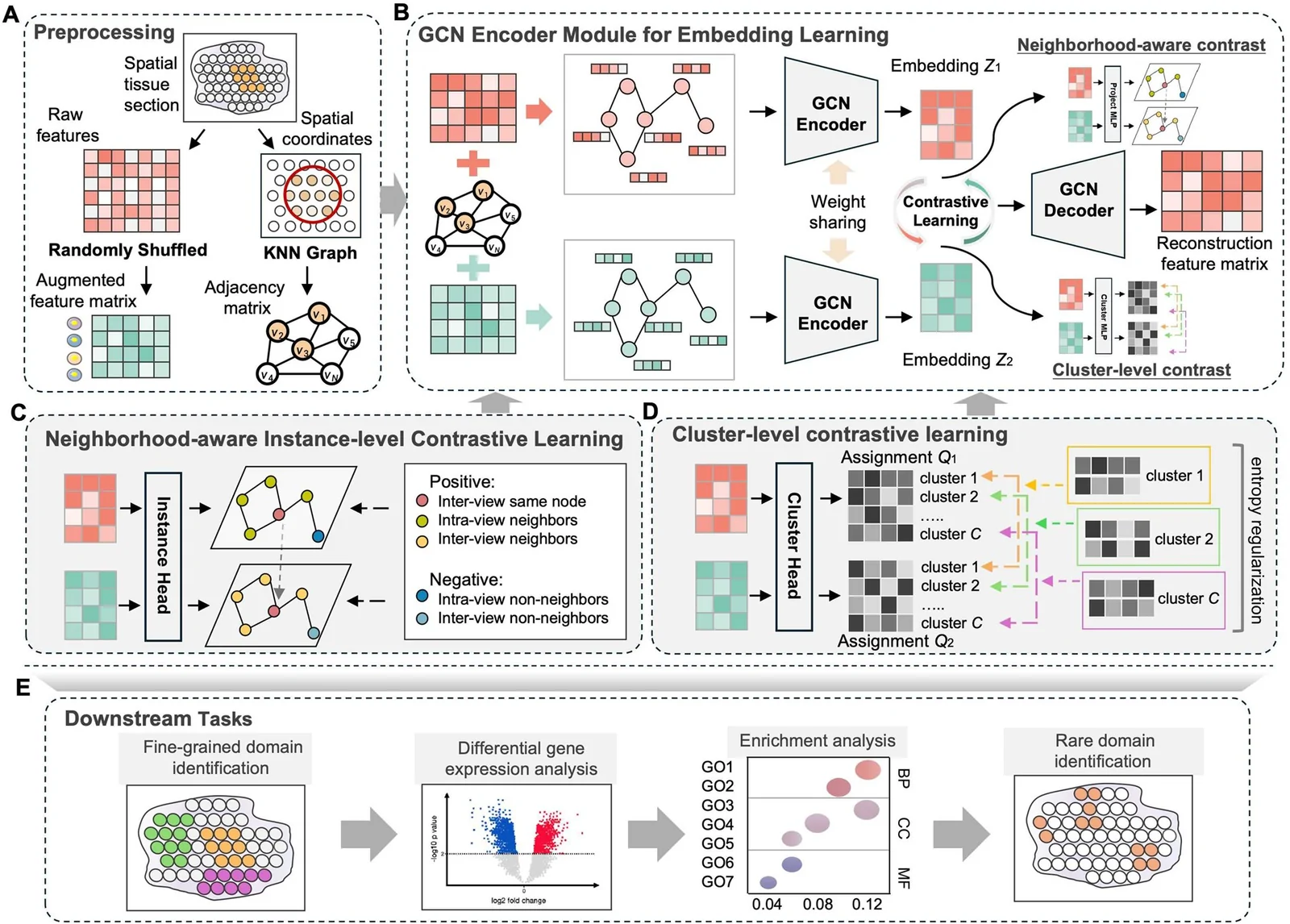
The overview of the proposed TRACER. (A) TRACER takes the gene expression matrix and spatial coordinates as input, constructs a K-nearest-neighbor spatial graph, and generates an augmented view by shuffling node feature vectors while preserving the graph topology. (B) The original and augmented views are passed through a shared graph convolutional autoencoder that learns latent spot representations from the expression matrix and graph adjacency matrix and reconstructs gene expression profiles via a reconstruction loss. (C) The neighborhood-aware instance-level contrastive module treats the same spot and its spatial neighbors across views as positive samples, thereby preserving local continuity. (D) The cluster-level contrastive module aligns cluster assignments across views and uses entropy regularization to enhance domain separability. (E) The reconstructed gene expression is used for spatial domain identification and downstream analyses, including differential expression analysis, functional enrichment analysis, and rare-domain interpretation.

### 2.2 Data preprocessing

In TRACER, the raw gene expression matrix was first processed using a standard *Scanpy* workflow. Low-quality or uninformative genes were removed where applicable, followed by library-size normalization, logarithmic transformation, and scaling to zero mean and unit variance. To reduce noise and computational cost, highly variable genes (HVGs) were selected as input features. For high-throughput platforms such as 10× Visium, the top 3000 HVGs were retained. For targeted or imaging-based platforms with limited gene coverage, including osmFISH, STARmap, and BARISTAseq, all measured genes were retained. The resulting expression matrix is denoted as $X\in R^{N\times M}$, where *N* is the number of spots and *M* is the number of selected genes.

### 2.3 Spatial neighborhood graph construction

For each tissue section, the spatial neighborhood graph *G* = (*V*, *E*) is constructed, where $V=\{v_{1},v_{2},\ldots \;,v_{N}\}$ denotes the set of N spot nodes, and E⊆(V×V) represents the set of edges between nodes. Pairwise Euclidean distances were computed from the spatial coordinates, and a *K*-nearest-neighbor strategy was used to define the adjacency matrix $A\in \{0,1{\}}^{N\times N}$; Specifically, $A_{ij}=1$ if spot *j* is among the *K* nearest neighbors of spot *i*, and $A_{ij}=0$ otherwise. Unless otherwise specified, *K* was set to 3 in all experiments; The KNN graph was symmetrized before model training. In TRACER, the adjacency matrix is used not only for graph convolutional message passing but also for defining spatial neighbor sets in the neighborhood-aware instance-level contrastive learning module.

### 2.4 Graph convolutional autoencoder backbone

TRACER employs a graph convolutional autoencoder as the backbone for learning spot representations from gene expression profiles and spatial neighborhood structure. Given the preprocessed expression matrix *X* and adjacency matrix *A*, self-loops are added to *A*, and the resulting matrix is symmetrically normalized as $\tilde{A}=D^{-1/2}(A{+I)D}^{-1/2}$, where *D* is the degree matrix of *A *+* I*. The encoder maps *X* into a low-dimensional latent representation *Z* through a graph convolutional layer:


(1) $$Z=\sigma (\tilde{A}XW_{e}+B_{e}),$$

where $Z\in R^{N\times d}$, *d* is the embedding dimension and σ(·) denotes the activation function. A symmetric graph convolutional decoder reconstructs the gene expression matrix from *Z*:


(2) $$H=\sigma (\tilde{A}ZW_{d}+B_{d}),$$

where $H\in R^{N\times M}$ denotes the reconstructed gene expression matrix. The reconstruction loss is defined as the mean squared error between *H* and *X*:


(3) $$L_{\text{rec}}=\frac{1}{N}\sum_{i=1}^{N}∥H_{i}-X_{i}{∥}^{2}.$$

The graph autoencoder provides the backbone representation for subsequent neighborhood-aware instance-level contrastive learning and cluster-level contrastive learning.

### 2.5 Neighborhood-aware instance-level contrastive learning for local spatial continuity

In spatial transcriptomics, neighboring spots within the same local tissue structure often exhibit related transcriptional programs. This property is particularly important for fine-grained or low-abundance spatial domains, where only a limited number of spots are available and individual spot-level expression profiles can be noisy. Conventional instance-level contrastive learning typically treats only the same spot across two augmented views as a positive pair, while ignoring spatially adjacent spots that may share the same local tissue context. To better preserve local spatial continuity, TRACER expands the definition of positive pairs by incorporating spatial neighbors into the instance-level contrastive objective.

To construct augmented views, we applied a feature-level perturbation strategy based on global feature shuffling (Zhu *et al.* 2020). Specifically, the rows of the original feature matrix Xwere randomly permuted across all spots/nodes, while the original spatial graph topology was kept unchanged. Thus, gene-expression feature vectors were globally reassigned among spots, whereas the coordinate-based KNN graph and its spatial adjacency relationships remained fixed. This feature-shuffled view was used only as a feature-level data augmentation strategy to construct a corrupted view for self-supervised contrastive learning, and was not intended to represent a biologically realistic tissue state. Therefore, TRACER does not assume that the shuffled feature assigned to a given spatial location is biologically similar to the original expression profile of that spot, nor does it define positive pairs according to the source location from which the shuffled feature is drawn. For clarity, the original graph is denoted as $G_{1}$, and the feature-shuffled augmented graph is denoted as $G_{2}$.

For the two views $G_{1}$and $G_{2}$, the latent representations of nodes are obtained via the encoder and denoted as $z_{u_{i}}$ and $z_{v_{i}}$, respectively. To enhance the expressive power of contrastive learning and decouple the encoder output space from the contrastive optimization space, we further introduce an instance-level projection head ϕ(⋅), which maps node representations into a contrastive space for similarity computation. Specifically, the projection head is implemented as a two-layer MLP with Layer Normalization and a ReLU activation function between the two linear transformations. The mapping is defined as:


(4) $$h_{i}=\phi (z_{i})=W_{2}^{I}\cdot \sigma (LN(W_{1}^{I}z_{i}+b_{1}^{I}))+b_{2}^{I},$$

where LN(⋅) denotes Layer Normalization, σ(⋅) is the ReLU activation function, and $W_{1}^{I}$, $W_{2}^{I}$, $b_{1}^{I}$, and $b_{2}^{I}$ are learnable parameters. The similarity function $\theta \left(\cdot ,\cdot \right)$ used in contrastive learning is computed based on the projected representations h, such as cosine similarity.

The definition of positive pairs is a critical design choice in contrastive learning for spatial transcriptomics. In GraphST and several related methods (Yu *et al.* 2026), each spot and its spatial neighbors are generally encouraged to have similar embeddings, thereby exploiting spatial adjacency as a prior to enhance local smoothness. TRACER shares this motivation, but extends the positive-pair definition by jointly considering instance correspondence across augmented views and spatial neighborhood relationships in the original coordinate-based KNN graph. Specifically, when constructing positive pairs, the representation of node $u_{i}$in $G_{1}$is treated as the anchor. The positive samples for anchor $u_{i}$ include three components: (i) the corresponding node $v_{i}$ in the other view, which forms a cross-view instance-level positive pair; (ii) its neighboring nodes within $G_{1}$, i.e. $u_{j}\in N_{i}^{1}$, which form intra-view neighbor positive pairs, where $N_{i}^{1}$ denotes the neighbor set of node $u_{i}$in the original KNN graph; and (iii) the neighboring nodes of $v_{i}$ in $G_{2}$, i.e. $v_{j}\in N_{i}^{2}$, which form cross-view neighbor positive pairs, where $N_{i}^{2}$ denotes the neighbor set of node $v_{i}$under the same fixed spatial graph topology.

This design differs from strategies that rely solely on spatial adjacency. The cross-view instance-level positive pair encourages representation consistency under feature-level perturbation, whereas the neighbor-based positive pairs preserve local tissue-context information and spatial continuity. In addition, non-positive nodes are not included in the spatial positive set and serve as candidate negative samples in the contrastive objective, which helps prevent unrelated or spatially distant spots from becoming overly close in the embedding space.

Thus, the total number of positive samples for the anchor $u_{i}$ is $N_{i}^{+}=|N_{i}^{1}|+|N_{i}^{2}|+1$, and all remaining nodes are treated as negative samples.

Based on this, the contrastive loss for anchor $u_{i}$ is defined as:


(5) l(ui)=-log⁡(f1,2(i,i)+∑uj∈Ni1f1,1(i,j)+∑vj∈Ni2f1,2(i,j))/N^if1,2(i,i)+∑(j≠i)(f1,1(i,j)+f1,2(i,j)),

where $f^{1,1}(i,j)=\text{exp}\;(\theta (u_{i},u_{j})/\tau )$, $f^{1,2}(i,j)=\text{exp}\;(\theta (u_{i},v_{j})/\tau )$, $f^{1,2}(i,i)=\text{exp}\;(\theta (u_{i},v_{i})/\tau )$, and θ(⋅,⋅) denotes cosine similarity, while τ is the temperature parameter (set to 0.2). The notation “1,1” refers to intra-view pairs, and “1,2” refers to inter-view pairs. The denominator includes both positive and negative pairs, which can be further decomposed as:


(6) $$\sum_{\left(j\neq i\right)}f^{1,1}(i,j)=\underset{\mathrm{intra}-\mathrm{view}\;\mathrm{neighbor}\;\mathrm{pos}\;}{\underset{⇓}{\sum_{u_{j}\in N_{i}^{1}}f^{1,1}(i,j)}}+\underset{\mathrm{intra}-\mathrm{view}\;\mathrm{neg}\;}{\underset{⇓}{\sum_{u_{j}\notin N_{i}^{1}}f^{1,1}(i,j)}}.$$

(7) $$\sum_{\left(j\neq i\right)}f^{1,2}(i,j)=\underset{\mathrm{inter}-\mathrm{view}\;\mathrm{neighbor}\;\mathrm{pos}\;}{\underset{⇓}{\sum_{v_{j}\in N_{i}^{2}}f^{1,2}(i,j)}}+\underset{\mathrm{inter}-\mathrm{view}\;\mathrm{neg}\;}{\underset{⇓}{\sum_{v_{j}\notin N_{i}^{2}}f^{1,2}(i,j)}}.$$

Let $u_{i}\;$and $v_{i}\;$ denote the latent representations of the same spatial spot i  obtained from two augmented views, respectively. Here, $u_{i}$ and $v_{i}$ are paired representations of spot iacross two views, rather than two adjacent spots or the two endpoints of a spatial graph edge. For each spot i, we compute the instance-level contrastive loss in two symmetric directions, using $u_{i}$ and $v_{i}$ as the anchor representation, respectively. Spatial adjacency information is incorporated only through the construction of the positive sample set, rather than by summing over graph edges. The final instance-level contrastive loss is defined as:


(8) $$L_{\mathrm{loc}-\text{cos}\;}=\frac{1}{2N}\sum_{i=1}^{N}\left[l(u_{i})+l(v_{i})\right],$$

where N is the number of spots.

By incorporating spatial neighbors into the positive set, this loss encourages spots within the same local tissue context to remain close in the embedding space. In contrast to methods that use spatial information only to construct the graph, TRACER uses the neighborhood structure as an explicit supervision signal during contrastive optimization. This design improves the stability of local representations and helps preserve fine-grained spatial structures that may otherwise be fragmented or absorbed by neighboring large domains.

### 2.6 Cluster-level contrastive learning for domain separability

Neighborhood-aware instance-level contrastive learning encourages local spatial continuity by pulling neighboring spots closer in the embedding space. However, local continuity alone may be insufficient for spatial domain identification, because adjacent domains with subtle transcriptional differences may be overly smoothed, particularly when small domains are embedded within larger tissue regions. To enhance domain-level separability, TRACER introduces cluster-level contrastive learning (Li *et al.* 2021) to align cluster assignments across augmented views and to encourage distinct cluster organization in the latent space.

Similar to the instance-level projection head ϕ(⋅), we introduce a cluster-level projection head ψ(⋅). This projection head is implemented as a two-layer multilayer perceptron (MLP), with Layer Normalization and a ReLU activation function inserted between the two linear transformations, followed by a Softmax function to output the probability distribution over clusters for each node. For the latent representation $z_{i}$ of node i, the cluster assignment probability vector is defined as:


(9) $$c_{i}=\psi (z_{i})=\mathrm{Softmax}(W_{2}^{C}\cdot \sigma (LN(W_{1}^{C}z_{i}+b_{1}^{C}))+b_{2}^{C}),$$

where $c_{i}\in R^{K}$ denotes the cluster assignment probability vector for node i, K is the number of clusters (i.e. *n_clusters*), LN(⋅) denotes Layer Normalization, σ(⋅) is the ReLU function, and $W_{1},W_{2},b_{1},b_{2}$ are learnable parameters.

For the two views $G_{1}$ and $G_{2}$, we obtain cluster assignment matrices $C^{\left(1\right)},C^{\left(2\right)}\in R^{N\times K}$, where N is the number of nodes (spots). By transposing these matrices, we derive cluster-level representations: $Q^{\left(1\right)}=(C^{\left(1\right)}{)}^{⊤}\in R^{K\times N},Q^{\left(2\right)}=(C^{\left(2\right)}{)}^{⊤}\in R^{K\times N}$. Where $q_{k}^{\left(1\right)}\in R^{N}$ represents the cluster-level representation of the k-th cluster in view $G_{1}$ (i.e. the probability vector of all nodes belonging to cluster k), and similarly for $q_{k}^{\left(2\right)}$ in view $G_{2}$. We denote:


(10) $$Q^{\left(1\right)}=\{q_{1}^{\left(1\right)},\ldots ,q_{K}^{\left(1\right)}\},Q^{\left(2\right)}=\{q_{1}^{\left(2\right)},\ldots ,q_{K}^{\left(2\right)}\}.$$

The similarity between cluster-level representations is defined as:


(11) $$s\left(q_{a}^{\left(v\right)},q_{b}^{\left(w\right)}\right)=\frac{\;\text{cos}\;\left(q_{a}^{\left(v\right)},q_{b}^{\left(w\right)}\right)}{\tau },$$

where τ is the temperature parameter (set to 0.5).

For each cluster k, the corresponding clusters across views form positive pairs: $\left(q_{k}^{\left(1\right)},q_{k}^{\left(2\right)}\right)$. Taking $q_{k}^{\left(1\right)}$ as the anchor, the contrastive loss is defined as:


(12) $$l_{k}^{\left(1\right)}=-\log\frac{\;\text{exp}\;\left(s(q_{k}^{\left(1\right)},q_{k}^{\left(2\right)})\right)}{\sum_{m=1}^{K}\;\text{exp}\;(s(q_{k}^{\left(1\right)},q_{m}^{\left(2\right)}))+\sum_{m\neq k}\;\text{exp}\;(s(q_{k}^{\left(1\right)},q_{m}^{\left(1\right)}))}.$$

Similarly, taking $q_{k}^{\left(2\right)}$as the anchor:


(13) $$l_{k}^{\left(2\right)}=-\log\frac{\;\text{exp}\;\left(s(q_{k}^{\left(2\right)},q_{k}^{\left(1\right)})\right)}{\sum_{m=1}^{K}\;\text{exp}\;(s(q_{k}^{\left(2\right)},q_{m}^{\left(1\right)}))+\sum_{m\neq k}\;\text{exp}\;(s(q_{k}^{\left(2\right)},q_{m}^{\left(2\right)}))}.$$

The final cluster-level contrastive loss is:


(14) $$L_{\text{cl}u}=\frac{1}{2K}\sum_{k=1}^{K}\left(l_{k}^{\left(1\right)}+l_{k}^{\left(2\right)}\right).$$

This objective treats the same cluster across two views as a positive pair, and different clusters, whether within the same view or across views, as contrastive negatives.

However, relying solely on cluster-level contrastive learning may lead to degenerate solutions, such as assigning all nodes to a few clusters or even a single cluster (i.e. cluster collapse). To avoid degenerate solutions in which most spots are assigned to a small number of clusters, we introduced an entropy-based regularization term on the marginal cluster distribution. This term encourages sufficient usage of cluster prototypes while preventing cluster collapse.

Specifically, for each view v∈{1,2}, let $C^{\left(v\right)}\in R^{N\times K}$ be the cluster assignment matrix. The marginal probability of cluster k is computed as:


(15) $$p_{k}^{\left(v\right)}=\frac{1}{N}\sum_{i=1}^{N}c_{ik}^{\left(v\right)},k=1,\ldots ,K,$$

where $c_{ik}^{\left(v\right)}$ denotes the probability that node iis assigned to cluster kin view v. This yields the marginal distribution vector: $p^{\left(v\right)}=[p_{1}^{\left(v\right)},\ldots ,p_{K}^{\left(v\right)}]$.

The negative entropy regularization term is then defined as:


(16) $$R_{\text{ent}}=\sum_{v\in \{1,2\}}\left(\log K+\sum_{k=1}^{K}p_{k}^{\left(v\right)}\;\text{log}\;p_{k}^{\left(v\right)}\right),$$

where log⁡K is a constant term ensuring that the regularization term reaches its minimum when cluster usage is non-degenerate, thereby reducing the risk that all spots collapse into one or a few clusters.

Finally, the overall cluster-level training objective is defined as:


(17) $$L_{\text{cluster}}=L_{\text{clu}}+\lambda \;R_{\text{ent}},$$

where λ is a tradeoff coefficient (set to 1 by default in this work). Together with the neighborhood-aware instance-level contrastive loss, the cluster-level objective allows TRACER to balance local spatial smoothness and domain-level discrimination. This balance is important for detecting fine-grained spatial domains, where the model must preserve local tissue continuity while avoiding the merging of subtle but functionally distinct regions. In summary, The overall training objective is defined as


(18) $$L=\alpha L_{\mathrm{rec}}+\beta L_{\mathrm{loc}-\cos}+\gamma L_{\mathrm{cluster}}$$

where α, β, and γ  are the hyperparameters to control the influence across the objective functions. Based on the parameter sensitivity analysis, α, β, and γ were set to 10, 0.2, and 0.5, respectively.

### 2.7 Spatial domain identification and downstream characterization

After training, TRACER used the reconstructed expression representation H for spatial domain identification. This representation is obtained from the contrastively optimized latent space and preserves both gene expression patterns and spatially constrained structure. PCA was applied to reduce dimensionality, followed by *Mclust* clustering to infer spatial domains.

For benchmark datasets with manual annotations, the number of clusters was set to the number of annotated domains to enable quantitative comparison with existing methods. For analyses designed to explore fine-grained local heterogeneity, a larger number of clusters was used to identify potential substructures within broadly annotated regions. To reduce the risk of interpreting arbitrary over-clustering as biologically meaningful structure, candidate fine-grained domains were further evaluated using cross-resolution stability analysis, domain-level correlation analysis, differential expression analysis, and Gene Ontology enrichment analysis. Therefore, these fine-grained results were interpreted as exploratory evidence of local molecular heterogeneity rather than definitive biological annotations.

A local spatial refinement strategy was applied to reduce isolated noisy labels. For each spot, neighboring spots within a fixed circular radius were identified, and the spot label was reassigned to the most frequent label among its neighbors. Under the default setting, the neighborhood of each spot was defined as a circular region centered on that spot with a radius of 50 in the original spatial coordinate system after preprocessing. This value represents a distance threshold in the processed spatial coordinates rather than a physical distance in micrometers. The refinement step was used to improve spatial coherence while preserving the overall clustering structure inferred from the learned representations. Spatial domains were visualized using Scanpy based on the original spatial coordinates. Detailed implementations of the downstream tasks are provided in File 1, available as supplementary data at *Bioinformatics* online.

### 2.8 Parameter settings and evaluation metrics

TRACER and all baseline methods were re-run under the same computational environment and evaluated using a unified evaluation pipeline. For all baseline methods, we followed the recommended workflows and parameter settings provided in the original publications and official code tutorials whenever available. TRACER was implemented in Python using PyTorch. In all experiments, the same default configuration was used, including a single-layer graph convolutional autoencoder, fixed loss weights, and fixed training epochs. Detailed software versions, default hyperparameters, optimizer settings, and baseline method configurations are provided in File 2, available as supplementary data at *Bioinformatics* online. In addition, TRACER was evaluated by Adjusted Rand Index (ARI) and Normalized Mutual Information (NMI); the definitions of benchmarking evaluation metrics are provided in File 3, available as supplementary data at *Bioinformatics* online.

### 2.9 Dataset description

We evaluated TRACER on multiple publicly available spatial transcriptomics datasets covering diverse platforms, species, and tissue types. These included sequencing-based and imaging-based spatial transcriptomics technologies, human and mouse samples, and both normal and disease-related tissues, such as brain and breast cancer. Detailed information on these datasets, including platform, tissue type, sample size, gene number, and access links, is provided in File 4 and Table 1, available as supplementary data at *Bioinformatics* online.

## 3 Results

### 3.1 TRACER precisely identifies spatial domains in complex brain tissue datasets

To evaluate the ability of TRACER to recover spatial domains in tissues with complex organization, we first applied it to the human dorsolateral prefrontal cortex (DLPFC) dataset generated by the 10× Visium platform (Maynard *et al.* 2021). This dataset contains manually annotated cortical layers and white matter regions and is widely used for benchmarking spatial domain identification methods. TRACER was compared with ten representative methods, including GraphST, stDCL, DenoiseST (Cui *et al.* 2024), SEDR, BayesSpace, STAGATE, STMIGCL (Ren *et al.* 2025), SpaGCN, stLearn, and Mclust. Across the DLPFC slices, TRACER achieved the highest overall ARI and NMI distributions, indicating stronger agreement with manual annotations than the competing methods (Fig. 2A).

**Figure 2:**
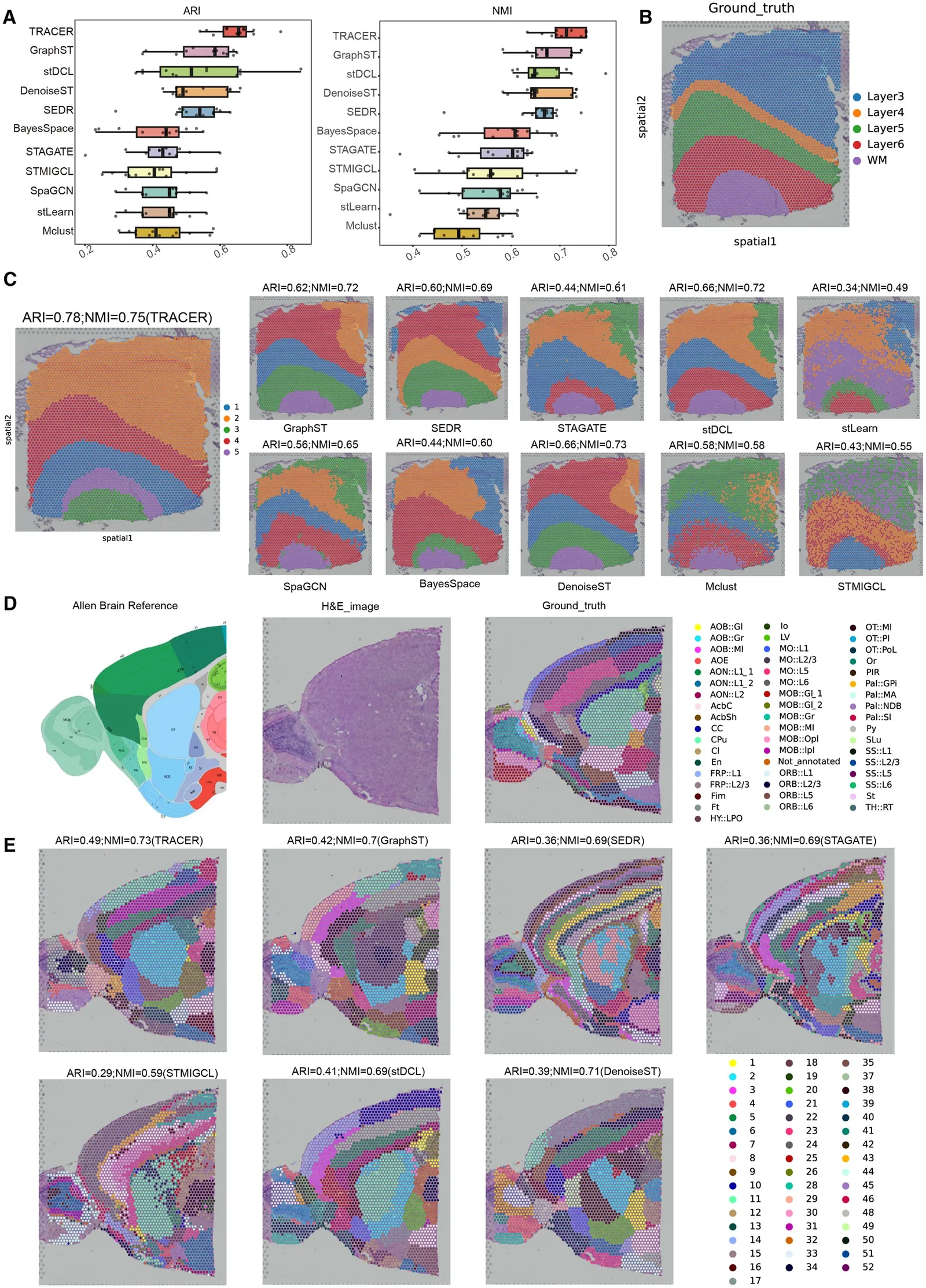
Spatial domain identification performance of TRACER on complex brain tissue datasets. (A) Comparison of TRACER and benchmark methods on the human dorsolateral prefrontal cortex (DLPFC) dataset using ARI and NMI metrics. (B) Ground-truth annotation of a representative DLPFC slice. (C) Spatial domain identification results of TRACER and benchmark methods on the representative DLPFC slice. The ARI and NMI scores are shown for each method. (D) Manual annotation of anatomical regions in the mouse anterior brain dataset based on the Allen Mouse Brain Atlas. (E) Spatial clustering results of TRACER and competing methods on the mouse anterior brain dataset.

We next examined a representative DLPFC slice to assess whether the quantitative improvement corresponded to spatially coherent domain recovery. TRACER produced domains that closely matched the annotated cortical layers and white matter, achieving an ARI of 0.78 and an NMI of 0.75 (Fig. 2B and C). Compared with competing methods, TRACER better preserved the continuity of laminar structures and reduced local domain mixing or fragmentation. Spatial domain identification results for the remaining DLPFC slices are provided in Figs 1–3, available as supplementary data at *Bioinformatics* online.

**Figure 3:**
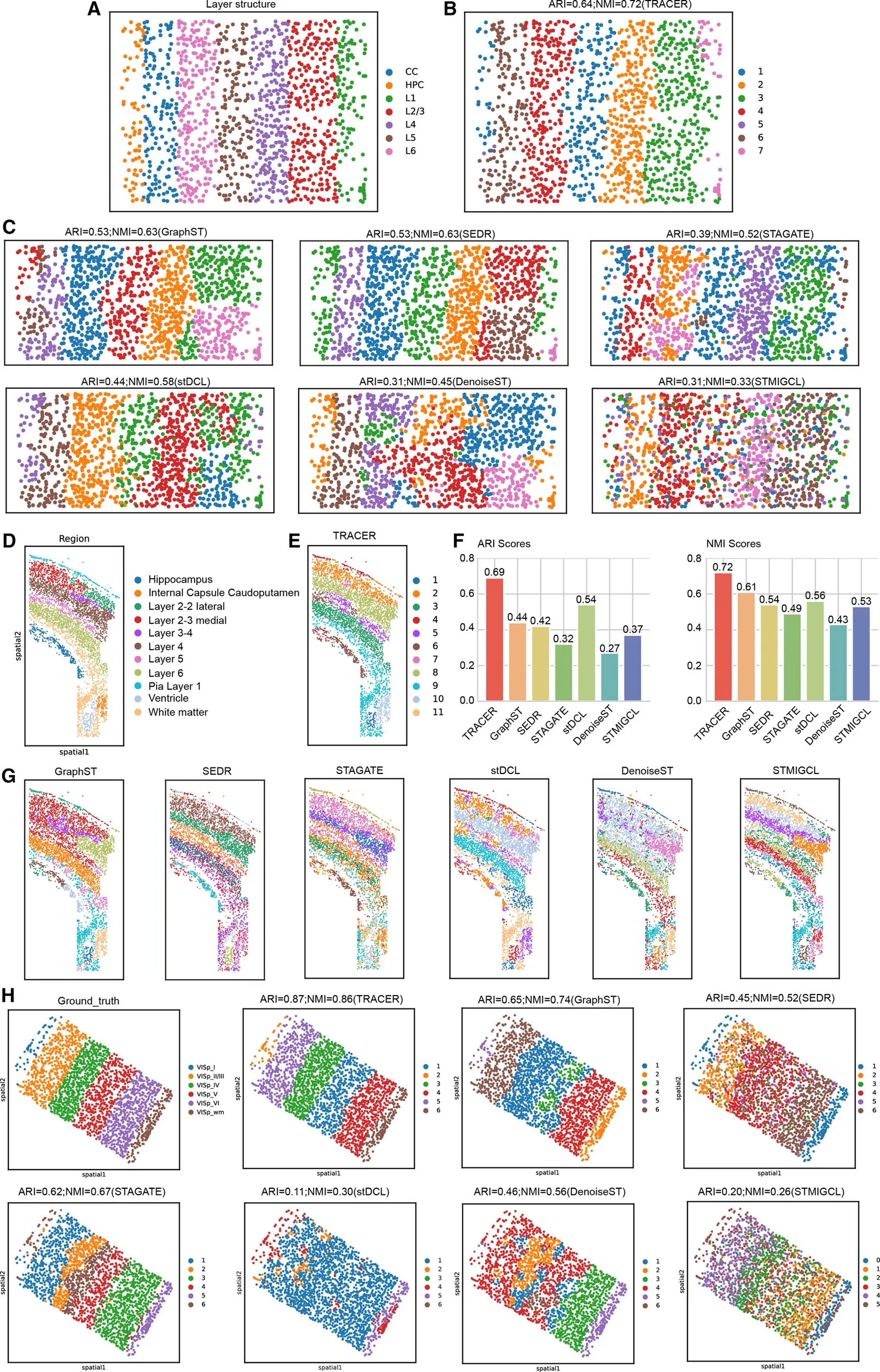
TRACER accurately delineates laminar and anatomical structures in mouse brain tissues. (A) Ground-truth annotation of the mouse primary visual cortex dataset. (B) Spatial domain identification result of TRACER on the mouse primary visual cortex dataset. (C) Spatial clustering results of benchmark methods on the mouse primary visual cortex dataset. (D) Ground-truth annotation of the mouse somatosensory cortex dataset. (E) Spatial domain identification result of TRACER on the mouse somatosensory cortex dataset. (F) Quantitative comparison of TRACER and benchmark methods on the mouse somatosensory cortex dataset using ARI and NMI. (G) Spatial clustering results of benchmark methods on the mouse somatosensory cortex dataset. (H) Spatial domain identification results of TRACER and benchmark methods on the BARISTAseq mouse visual cortex dataset containing six annotated laminar domains.

To further test TRACER on a more complex anatomical structure, we applied it to the mouse anterior brain dataset, whose regions were manually annotated against the Allen Mouse Brain Atlas (Lein *et al.* 2007). This dataset contains more spatial domains and irregular anatomical boundaries than the DLPFC benchmark. TRACER achieved the best performance among the compared methods, with an ARI of 0.49 and an NMI of 0.73, and recovered major anatomical structures with higher spatial coherence than competing methods (Fig. 2D and E). However, the absolute ARI remains modest, indicating that spatial domain identification in this dataset is still challenging and that TRACER does not fully resolve all annotated anatomical structures. This may reflect weak transcriptomic differences between neighboring regions, continuous transitions in tissue organization, uncertainty in manual anatomical boundaries, or highly similar expression profiles across some domains. Together, these results show that TRACER performs robustly across both layered cortical tissue and anatomically complex brain regions, while also highlighting the need for further improvements on datasets with complex tissue organization and subtle inter-domain differences.

### 3.2 TRACER delineates laminar and anatomical structures in the mouse brain tissues

To further evaluate whether TRACER can recover biologically meaningful spatial organization across different spatial transcriptomics technologies, we applied it to three mouse brain spatially resolved transcriptomic datasets: mouse primary visual cortex generated by STARmap (Wang *et al.* 2018), mouse somatosensory cortex generated by osmFISH (Codeluppi *et al.* 2018), and mouse visual cortex dataset generated by BARISTAseq (Chen Xiaoyin *et al.* 2018). These datasets contain ordered cortical layers or annotated anatomical regions and therefore provide complementary benchmarks for assessing spatial domain identification across tissue structures and measurement platforms. TRACER was compared with six representative methods that perform better in the previous complex brain tissue datasets, including GraphST, SEDR, STAGATE, stDCL, DenoiseST, and STMIGCL.

In the STARmap mouse primary visual cortex dataset, TRACER accurately recovered the laminar organization of the visual cortex, including cortical layers and adjacent anatomical regions, and achieved the best performance among the compared methods, with an ARI of 0.64 and an NMI of 0.72 (Fig. 3A–C). In the osmFISH mouse somatosensory cortex dataset, which contains more irregular cortical and non-cortica anatomical regions, TRACER produced spatial domains that were highly consistent with manual annotations and achieved the highest ARI and NMI values among the evaluated methods (ARI = 0.69, NMI = 0.72; Fig. 3D–G). These results indicate that TRACER can recover both ordered laminar structures and more complex anatomical organizations.

We further evaluated TRACER on the BARISTAseq mouse visual cortex dataset, which contains three tissue sections with annotated VISp layers (primary visual cortex). On the section shown in Fig. 3H, TRACER achieved an ARI of 0.87 and an NMI of 0.86, outperforming the compared methods and accurately reconstructing the six annotated laminar domains, including VISp_I, VISp_II/III, VISp_IV, VISp_V, VISp_VI, and VISp_wm. Results on the two additional BARISTAseq sections are provided in Fig. 4, available as supplementary data at *Bioinformatics* online. Together, these analyses show that TRACER can delineate laminar and anatomical structures across spatially resolved mouse brain datasets generated by different experimental platforms.

**Figure 4:**
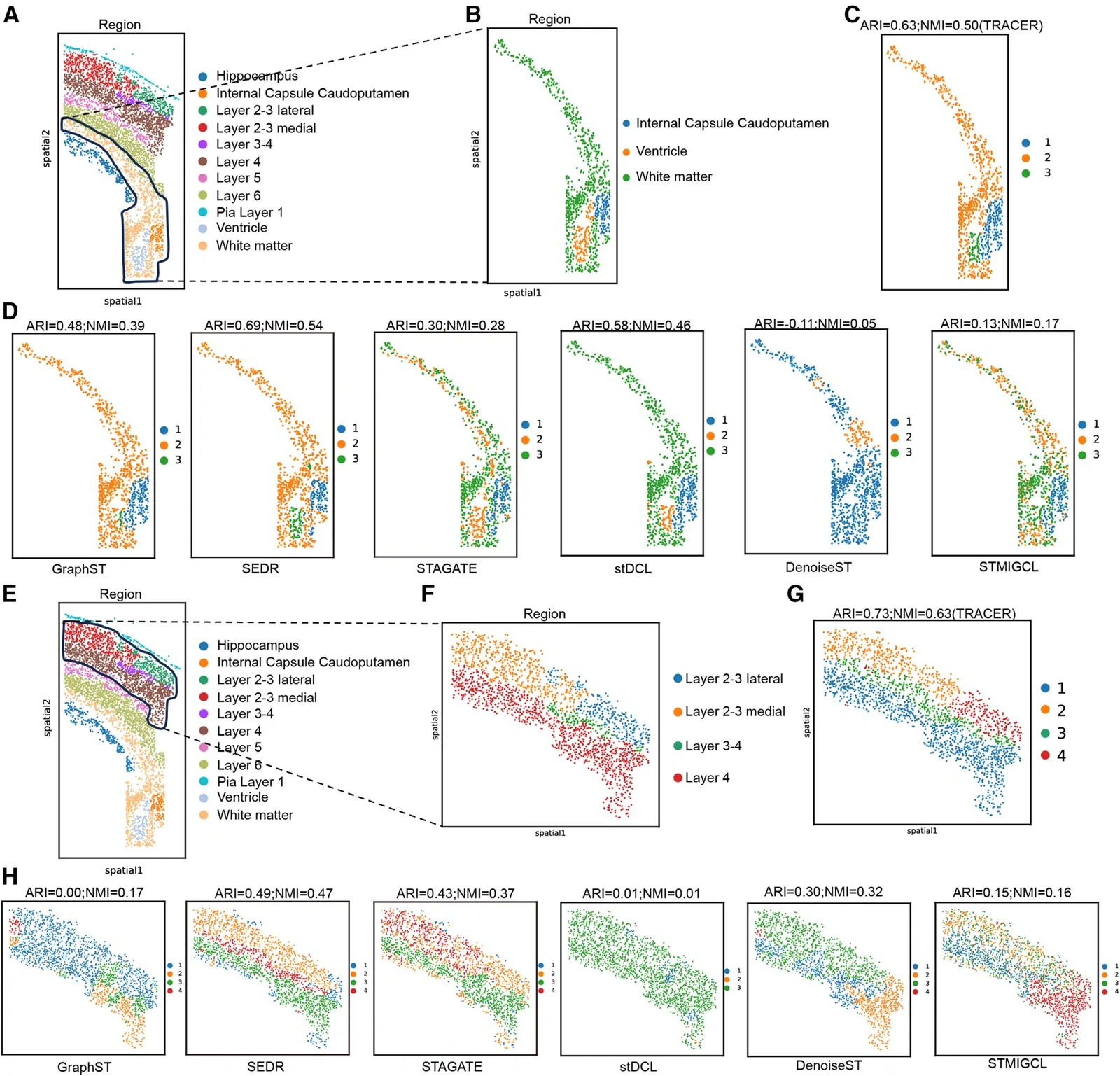
TRACER resolves rare spatial domains in the mouse somatosensory cortex. (A) Ground-truth annotation of selected spatial domains from the osmFISH mouse somatosensory cortex dataset. (B) Spatial visualization of the selected domains used for rare-domain identification analysis, including Ventricle, Internal Capsule–Caudoputamen, and white matter. (C) Quantitative comparison of TRACER and benchmark methods on the first rare-domain subset using ARI and NMI. (D) Spatial domain identification results of TRACER and benchmark methods on the first rare-domain subset. (E) Ground-truth annotation of the second selected subset, including Layer 3–4, Layer 2–3 lateral, Layer 2–3 medial, and Layer 4. (F) Spatial visualization of the selected cortical transition-layer domains. (G) Quantitative comparison of TRACER and benchmark methods on the second rare-domain subset using ARI and NMI. (H) Spatial domain identification results of TRACER and benchmark methods on the second rare-domain subset.

### 3.3 TRACER resolves rare spatial domains in the mouse somatosensory cortex

To evaluate the ability of TRACER to identify rare spatial domains, we performed rare-domain analyses using the osmFISH mouse somatosensory cortex dataset. In this study, rare domains were operationally defined as spatial domains accounting for less than 5% of the total spots in a given tissue section or analyzed subset. Because there is currently no universally accepted cutoff for defining rare spatial domains in spatial transcriptomics, we adopted 5% as an empirical threshold, following prior rare population detection studies such as GiniClust (Jiang *et al.* 2016), which defined rare cell clusters as populations comprising less than 5% of the total cells. Based on the manual annotations of the osmFISH dataset, Layer 3–4, Ventricle, and Internal Capsule–Caudoputamen accounted for 2.7%, 2.7%, and 2.2% of all spots, respectively, and therefore met our predefined criterion for rare domains. These three domains were selected for targeted evaluation of rare-domain identification performance (Fig. 5, available as supplementary data at *Bioinformatics* online).

**Figure 5:**
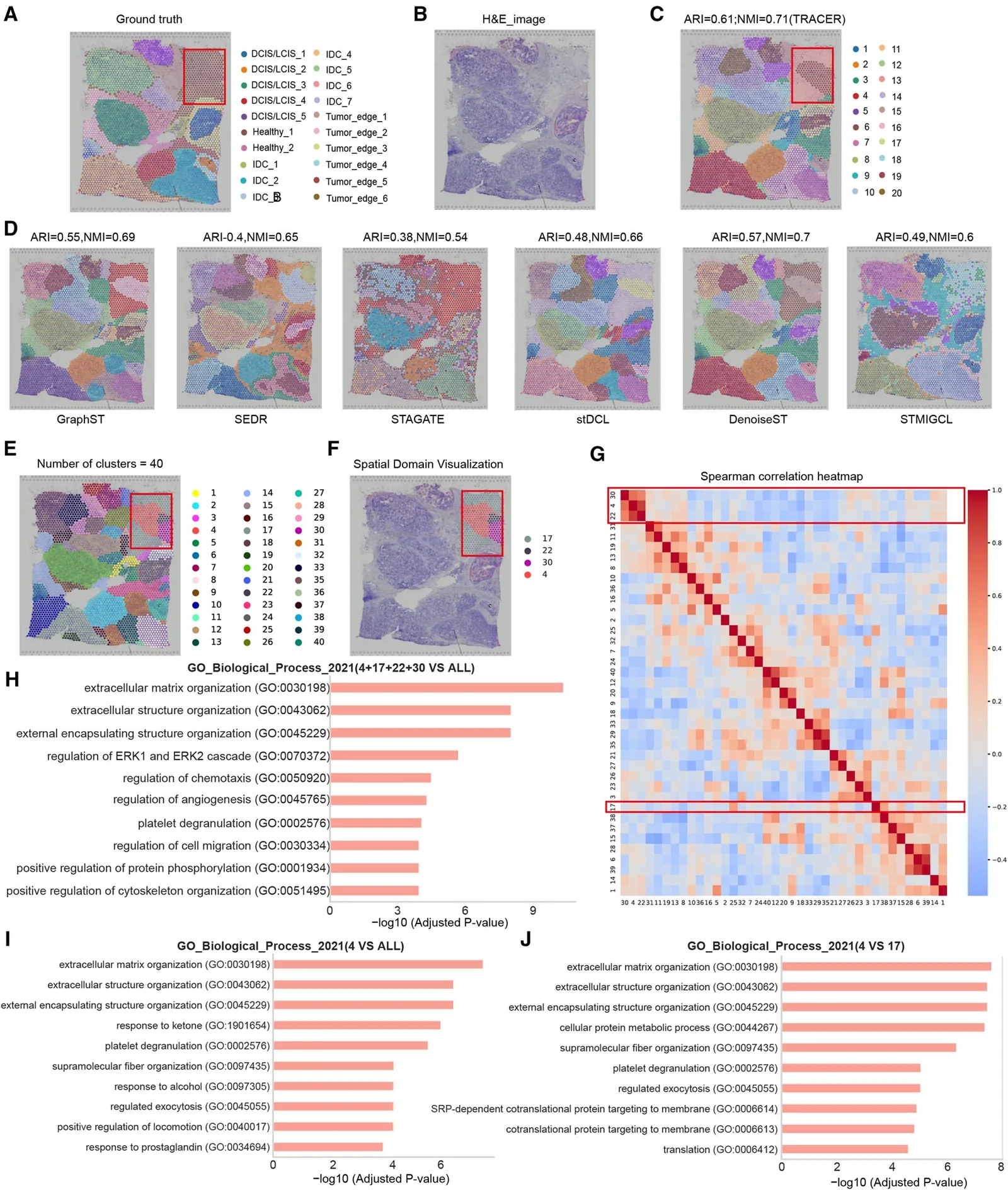
TRACER identifies small spatial domains with distinct functional programs in human breast cancer tissue. (A) Manual annotation of the human breast cancer spatial transcriptomics dataset. (B) Hematoxylin and eosin-stained image of the corresponding breast cancer tissue section.(C) Spatial domain identification result of TRACER on the breast cancer dataset. (D) Spatial clustering results of benchmark methods on the breast cancer dataset. (E) Fine-grained spatial domain identification results for the Healthy_1 region. (F) Enlarged visualization of the Healthy_1-associated region showing domains 4, 17, 22, and 30. Domains 4, 22, and 30 were defined as small spatial domains based on their low spot proportions. (G) Spearman correlation heatmap showing similarities among spatial domains. (H) Functional enrichment analysis of differentially expressed genes from domains 4, 17, 22, and 30. (I) GO enrichment analysis of domain 4 compared with all other spatial domains. (J) GO enrichment analysis of domain 4 compared specifically with domain 17.

We constructed two rare-domain-related subsets to assess different types of spatial organization. The first subset included Ventricle, Internal Capsule–Caudoputamen, and white matter, representing spatially adjacent or anatomically associated non-cortical regions (Fig. 4A and B). In this subset, TRACER achieved an ARI of 0.63 and an NMI of 0.50, which were slightly lower than those of SEDR (ARI = 0.69, NMI = 0.54) but higher than the remaining compared methods (Fig. 4C and D). These results suggest that TRACER performs comparably to the best-performing method in separating spatially adjacent rare non-cortical domains, while maintaining an advantage over most other compared methods. UMAP visualization of the learned embeddings further showed that TRACER produced a clear separation of rare-domain spots while preserving local spatial structure, providing qualitative support for its ability to learn discriminative representations for rare domains (Fig. 6, available as supplementary data at *Bioinformatics* online).

**Figure 6:**
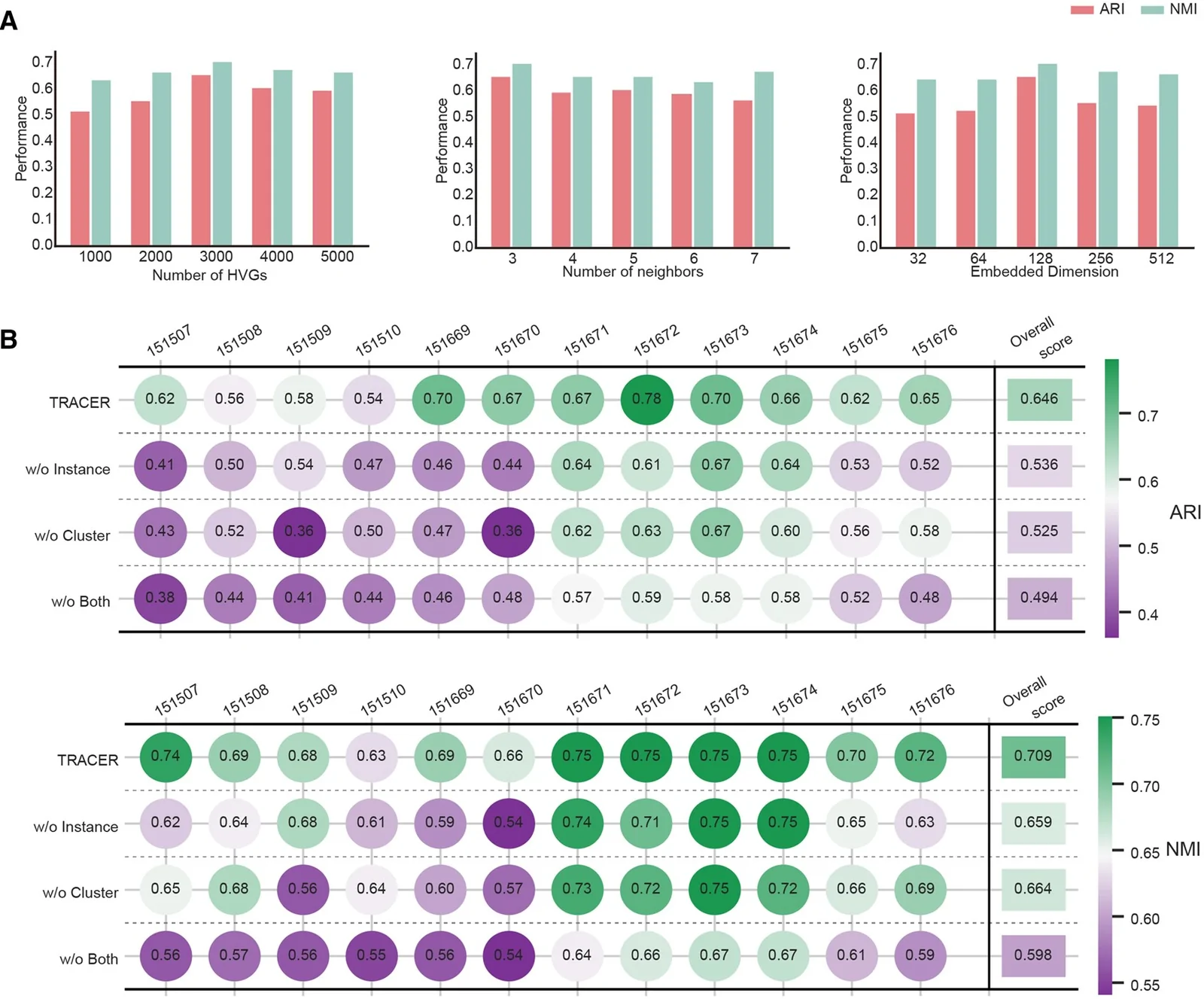
Robustness and ablation analysis of TRACER. (A) Parameter sensitivity analysis of TRACER on the DLPFC dataset. (B) Ablation analysis of TRACER. Three ablated variants were evaluated: TRACER without instance-level contrastive learning, TRACER without cluster-level contrastive learning, and TRACER without both contrastive learning components.

The second subset focused on a cortical transition structure, including Layer 3–4, Layer 2–3 lateral, Layer 2–3 medial, and Layer 4 (Fig. 4E and F). Layer 3–4 is positioned between Layer 2–3 and Layer 4, making it a useful test case for evaluating whether a method can preserve subtle hierarchical structure and distinguish adjacent cortical domains. In this subset, TRACER achieved the best performance, with an ARI of 0.73 and an NMI of 0.63, and more accurately recovered the spatial distribution of the four selected domains than the competing methods (Fig. 4G and H). UMAP visualization showed similarly separable latent structures for these adjacent cortical domains (Fig. 6, available as supplementary data at *Bioinformatics* online). This result suggests that TRACER may be particularly advantageous in identifying transitional layers, boundary regions, or spatial domains with subtle hierarchical organization.

Together, these results show that TRACER is competitive in rare-domain-related analyses and shows advantages in transitional-layer settings in the osmFISH mouse somatosensory cortex dataset. Although TRACER was not the top-performing method in all rare-domain scenarios, its competitive performance in the non-cortical subset and superior performance in the cortical transition-layer subset support its ability to capture fine- grained spatial heterogeneity in selected rare-domain-related and transitional-layer contexts.

### 3.4 TRACER identifies small spatial domains with distinct functional programs in human breast cancer tissue

We next evaluated whether TRACER could resolve fine-grained spatial heterogeneity in disease tissue using a 10× Visium human breast cancer dataset (Wu *et al.* 2021). This dataset contained one tissue section with 3798 spatial spots and 36 601 detected genes and was manually annotated into 20 spatial regions based on H&E staining and breast cancer-related marker genes, including invasive ductal carcinoma, ductal carcinoma in situ/lobular carcinoma in situ, normal tissue, and tumor border regions (Fig. 5A and B). Compared with representative benchmark methods, TRACER achieved the best spatial domain identification performance on this dataset, with an ARI of 0.61 and an NMI of 0.71 (Fig. 5C and D).

Inspection of the TRACER clustering results suggested that the upper-right region annotated as Healthy_1 may contain additional internal spatial heterogeneity. Because fine-grained clustering can be sensitive to the choice of cluster number K, we performed a cross-resolution stability analysis to evaluate whether the observed substructure was stable across different clustering resolutions. Specifically, we varied K from 15 to 45 while keeping the learned TRACER embedding fixed, and used clustree (Zappia and Oshlack 2018) to visualize the inheritance, splitting, and reorganization patterns of clusters across adjacent K values (Fig. 7, available as supplementary data at *Bioinformatics* online).

The clustree analysis showed that the Healthy_1-associated clusters followed continuous inheritance trajectories as K increased, without obvious large-scale cross-mixing or frequent reorganization. In particular, the subdomains observed at K = 40 could be traced from lower-resolution clustering results and remained largely stable when K was further increased. These results suggest that the fine-grained subdivisions within the Healthy_1 region are not solely driven by a single arbitrary choice of K, but instead show cross-resolution stability.

Based on this stability analysis, we selected the K = 40 as the result for exploratory downstream characterization. At K = 40, the Healthy_1-associated region was subdivided into four spatial domains: 4, 17, 22, and 30 (Fig. 5E and F). Among them, domains 4, 22, and 30 accounted for only 3.7%, 0.6%, and 1.4% of all spots, respectively, and were therefore considered small spatial domains in this analysis (Fig. 8, available as supplementary data at *Bioinformatics* online). Together, these results provide evidence of stable fine-grained spatial heterogeneity within the Healthy_1 region, although these subdomains should be interpreted as exploratory findings rather than definitive biological annotations in the absence of independent orthogonal validation.

We next examined whether these small spatial domains showed biologically interpretable molecular differences rather than being merely arbitrary over-clustering results. Spearman correlation analysis showed that domains 4, 22, and 30 were more similar to one another than to domain 17 (Fig. 5G). To further characterize the biological functions of these spatial domains, we performed pairwise differential expression analysis among domains 4, 17, 22, and 30. For each pairwise comparison, differentially expressed genes were separated according to the direction of regulation. Specifically, genes up-regulated in one domain relative to the compared domain were used for pathway enrichment analysis of that domain, whereas genes up-regulated in the other domain were analyzed separately. Therefore, pathway enrichment was conducted using domain-specific up-regulated genes rather than pooled differentially expressed genes.

Based on these domain-specific up-regulated genes, enriched terms for domains 4, 17, 22, and 30 were mainly associated with extracellular matrix organization, structural maintenance (Brassart-Pasco *et al.* 2020) and microenvironment remodeling (Figs 5H and Fig. 9, available as supplementary data at *Bioinformatics* online). Domain 17 showed a distinct functional profile based on its up-regulated genes, including protein metabolic modification, cell activation, motility, proliferation, and inflammatory signaling (Fig. 10, available as supplementary data at *Bioinformatics* online).

Further comparison of domains 4, 22, and 30 with all other domains and with domain 17 revealed domain-specific functional tendencies. Enrichment analysis of up-regulated genes showed that domain 4 was mainly associated with extracellular matrix organization and structural maintenance, domain 22 with translation, membrane transport, and secretory processing, and domain 30 with collagen-associated matrix remodeling and peptidase-related regulation (Wu *et al.* 2025), see details in Fig. 5I and J; Figs 9–16, available as supplementary data at *Bioinformatics* online. These results suggest that the broadly annotated Healthy_1 region contains multiple small spatial domains with distinct molecular programs, demonstrating TRACER’s ability to uncover fine-grained functional heterogeneity in complex tissue contexts. A detailed description of the results is provided in File 6, available as supplementary data at *Bioinformatics* online.

### 3.5 Robustness and efficiency analysis of TRACER

We further performed a series of sensitivity analyses, ablation experiments, robustness analyses, and computational evaluations to systematically assess the parameter robustness, component contribution, rare-domain identification ability, and scalability of TRACER. First, we evaluated the sensitivity of TRACER to key hyperparameters on the DLPFC dataset. Specifically, we examined the effects of the number of HVGs, the number of neighbors used for graph construction, and the embedding dimensionality. Following prior studies, we considered reasonable parameter ranges, including HVGs in [1000, 2000, 3000, 4000, 5000], the number of neighbors in [3, 4, 5, 6, 7], and embedding dimensions in [32, 64, 128, 256, 512]. The results showed that TRACER achieved the best performance with 3000 HVGs, 3 neighbors, and an embedding dimension of 128, whereas other parameter settings led to varying degrees of performance degradation (Fig. 6A). In addition, we evaluated the effects of the loss weights and the hyperparameter λ on model performance. These analyses showed that TRACER maintained relatively stable performance within a reasonable range of weighting parameters, although suboptimal weighting could reduce performance. Detailed results are provided in File 7 and Table 2, available as supplementary data at *Bioinformatics* online.

We next conducted ablation studies to assess the contribution of each major component in TRACER. The model consists of three key modules: a GCN-based autoencoder, instance-level contrastive learning, and cluster-level contrastive learning. To evaluate the importance of the two contrastive learning components, we designed three ablation settings: (i) removing the instance-level contrastive learning module (w/o Instance), (ii) removing the cluster-level contrastive learning module (w/o Cluster), and (iii) removing both contrastive learning modules while retaining only the GCN autoencoder backbone (w/o Both). On the DLPFC dataset, removing either contrastive component decreased performance, whereas removing both resulted in the most substantial performance drop, demonstrating that both instance-level and cluster-level contrastive learning contribute to the overall effectiveness of TRACER (Fig. 6B). Importantly, to directly evaluate whether the contrastive learning components contribute to the identification of rare spatial domains, we further performed ablation analysis on two rare domain datasets. Specifically, we compared the full TRACER model with the same ablation variant described above. The results show that removing either instance-level or cluster-level contrastive modules weakens TRACER’s ability to recover spatially coherent rare domains. In contrast, removing both contrastive modules simultaneously leads to the most significant performance degradation, with rare domains becoming difficult to distinguish, fragmented, or partially merged with neighboring domains. These results indicate that the two contrastive learning components not only contribute to overall clustering performance but also to the detection and preservation of rare spatial domains. The detailed rare-domain ablation results are provided in Fig. 17, File 8, and Tables 3 and 4, available as supplementary data at *Bioinformatics* online.

We also evaluated the influence of the post-processing strategy. Since TRACER applies a spatial refinement step after clustering, we compared its performance with and without this refinement procedure. This analysis was conducted on both the DLPFC dataset and the rare-domain datasets. The results showed that spatial refinement further improved the spatial coherence and clustering accuracy of the predicted domains. Nevertheless, even without post-processing, TRACER still maintained competitive performance. Detailed post-processing ablation results are provided in File 8 and Tables 5–7, available as supplementary data at *Bioinformatics* online.

We further examined the influence of model architecture and augmentation strategy. First, we evaluated different GCN depths and found that increasing the number of GCN layers led to decreased performance, supporting the use of a single-layer GCN encoder in TRACER. This may be because deeper GCNs tend to introduce excessive spatial smoothing, which can obscure fine-grained or rare spatial domain boundaries. Second, we compared different graph neural network backbones to assess whether TRACER depends on a specific GNN architecture. The results showed that the GCN-based encoder provided stable and competitive performance, supporting its use as the default backbone. Third, we evaluated different graph augmentation strategies, including feature shuffling and other commonly used augmentations. These results further confirmed that the augmentation design affects representation learning and downstream domain identification. Detailed results for GCN depth, GNN backbone, and augmentation strategy are provided in File 9 and Tables 8–10, available as supplementary data at *Bioinformatics* online.

To assess the robustness of TRACER to random initialization, we further tested the model using multiple random seeds in addition to the originally selected seed. The results showed that different random seeds caused only limited performance variation, indicating that TRACER maintains relatively stable performance under different initializations. Detailed results are provided in File 10 and Table 11, available as supplementary data at *Bioinformatics* online.

Finally, we evaluated the computational efficiency and scalability of TRACER. We first compared the runtime and peak GPU memory usage of TRACER with those of baseline methods on the DLPFC dataset (Fig. 18, File 11, and Table 12, available as supplementary data at *Bioinformatics* online). Although TRACER required longer runtime, likely due to the increased model complexity and the additional computational cost introduced by contrastive learning, it showed a clear advantage in terms of GPU memory consumption, indicating superior memory efficiency. We further assessed the scalability of TRACER by varying the number of spots from 1000 to 400 000 and recording the corresponding runtime and peak GPU memory usage. The results showed that TRACER remained applicable to large-scale spatial transcriptomics datasets, with computational costs increasing predictably as the number of spots increased. Detailed scalability results are provided in File 11 and Table 13, available as supplementary data at *Bioinformatics* online. Overall, these analyses demonstrate that TRACER achieves a favorable balance among performance, robustness, rare-domain detection ability, and computational efficiency, particularly in terms of memory usage.

## 4 Conclusion

In this study, we developed TRACER, a twin-contrastive learning framework for fine-grained and rare spatial domain identification in spatial transcriptomics. By integrating a graph convolutional autoencoder with neighborhood-aware instance-level contrastive learning and cluster-level contrastive learning, TRACER learns spatial representations that preserve local tissue continuity while maintaining domain-level separability. This design allows spatially adjacent spots to guide local representation learning and encourages robust separation of neighboring or low-abundance domains.

Across multiple spatially resolved transcriptomic datasets, TRACER showed strong performance in recovering major anatomical structures and finer local domains. It accurately delineated cortical layers and complex anatomical regions in human and mouse brain datasets, showed competitive performance in rare-domain-related analyses and superior performance in a cortical transitional-layer subset in the osmFISH mouse somatosensory cortex dataset, and identified small spatial domains with distinct molecular programs in human breast cancer tissue. Ablation analysis confirmed the complementary contributions of the two contrastive modules, while efficiency analysis indicated that TRACER provides a practical tradeoff between improved performance and computational cost.

TRACER still has several limitations. It currently uses only gene expression and spatial coordinates, and future extensions could incorporate histological images, protein measurements, or spatial multi-omics data. In addition, TRACER analyzes tissue sections independently, and multi-slice or multi-sample integration remains an important direction. Furthermore, although TRACER shows improved performance in identifying rare-domain-related structures, the detection of rare spatial domains can still be affected by their size, spatial continuity, and molecular separability, particularly when rare regions are extremely small or exhibit gradual transitions to surrounding tissues. Finally, TRACER requires a predefined clustering number K, and inappropriate choices of Kmay lead to over-partitioning or merging of biologically meaningful domains. Future work will focus on developing more adaptive strategies for determining Kand further improving the robustness of rare-domain detection. Overall, TRACER provides a useful computational framework for identifying major and fine-grained spatial domains, including selected rare-domain-related and transitional-layer contexts, and for dissecting local spatial heterogeneity in complex tissues.

## Supplementary Material

btag805_Supplementary_Data

## Data Availability

All datasets used in this study are publicly available from published studies or public databases. The corresponding data access links have been compiled and provided in File 1, available as supplementary data at *Bioinformatics* online.

## Code availability

The Python implementation of TRACER is available at https://github.com/luxin-heart/TRACER.
