# Supplementary material for: TRACER: twin-contrastive learning for fine-grained and rare spatial domain identification in spatial transcriptomics
Source: Bioinformatics. 2026 Oct 30;42(11):btag805. doi: 10.1093/bioinformatics/btag805 (PMC13746605; doi:10.1093/bioinformatics/btag805)
Supplement: Supplementary file 1 [file btag805_supplementary_data.docx]

**Supplementary Information for TRACER: Twin-contrastive learning for fine-grained and rare spatial domain identification in spatial transcriptomics**

Qiaoming Liu^1#^, Xin Lu^2,3#^, Bingsen Song^1^, Qianran Wei^4^, Wenzhe Feng^1^, Kexin Guo^1^, Jian Wang^5^, Murong Zhou^4^*, Yuming Zhao^4^*, Guohua Wang^2,3^*

^1^ School of Artificial Intelligence, Henan University, Zhengzhou 450000, China

^2^ Faculty of Computing, Harbin Institute of Technology, Harbin 150001, China

^3^ Zhengzhou Advanced Research Institute, Harbin Institute of Technology, Zhengzhou 450000, China

^4^ School of Computer Science and Artificial Intelligence, Northeast Forestry University, Harbin 150040, China

^5^ School of Computer and Information Engineering, Henan University of Economics and Law, Zhengzhou 450000, China

E-mail: ghwang@hit.edu.cn, zym@nefu.edu.cn, doriszmr@nefu.edu.cn

^#^: Qiaoming Liu and Xin Lu contributed equally to this research.

[1	Supplementary File 1: Details of the functional characterization of fine-grained spatial domains	3](#_Toc238454441)

[2	Supplementary File 2: Details of the parameter settings	5](#_Toc238454442)

[3	Supplementary File 3: Definition of benchmarking evaluation metrics	6](#_Toc238454443)

[4	Supplementary File 4: Dataset descriptions and data access links	7](#_Toc238454444)

[5	Supplementary File 5: The Supplementary figures.	8](#_Toc238454445)

[6	Supplementary File 6: Details of the analysis results of small spatial domains on human breast cancer tissue	25](#_Toc238454446)

[7	Supplementary File 7: Parameter analysis of TRACER	30](#_Toc238454447)

[8	Supplementary File 8: Ablation experiments of TRACER	31](#_Toc238454448)

[9	Supplementary File 9: Architecture and Augmentation Sensitivity Analysis	34](#_Toc238454449)

[10	Supplementary File 10: Random Seed Robustness Analysis	36](#_Toc238454450)

[11	Supplementary File 11: Computational Efficiency and Scalability Analysis	37](#_Toc238454451)

1 **Supplementary File 1: Details of the functional characterization of fine-grained spatial domains**

To assess whether the fine-grained spatial domains identified by TRACER correspond to biologically meaningful subregions rather than arbitrary over-clustering, we performed domain-level correlation analysis, differential expression analysis, and functional enrichment analysis.

**(i) Differential gene expression analysis**

To identify key genes distinguishing spatial domains, differential gene expression analysis is performed using the sc.tl.rank_genes_groups() function in *Scanpy* with the Wilcoxon rank-sum test. Genes are considered significantly differentially expressed if they satisfy the following criteria: p-value < 0.05 and |log₂ fold change| > 1. The resulting differentially expressed genes (DEGs) are retained for downstream functional enrichment analysis.

**(ii) Correlation analysis**

To quantify the similarity among spatial domains, we perform correlation analysis based on cluster-level representations. For each domain, a centroid is computed by averaging the feature vectors of all spots within the domain, using PCA-reduced embeddings (adata.obsm['X_pca']) to obtain a denoised and compact representation of gene expression profiles. Pairwise similarities between domains are then measured using the Spearman rank correlation coefficient, resulting in a domain–domain correlation matrix. Compared with Pearson correlation, Spearman correlation is more robust to non-linear relationships and outliers, making it well-suited for biological data.

To further characterize the relationships among domains, hierarchical clustering with average linkage is applied to the correlation matrix. The resulting dendrogram is used to reorder the matrix, grouping domains with higher similarity. The reordered correlation matrix is visualized as a heatmap using Seaborn, where color intensity reflects the strength and direction of correlations. This analysis reveals global patterns of similarity and potential functional relationships among spatial domains.

**(iii) Functional enrichment analysis**

To investigate the biological functions associated with the identified DEGs, Gene Ontology (GO) enrichment analysis is performed using *GSEApy* via the Enrichr interface (gp.enrichr). The input gene list consists of DEGs from each spatial domain. Enrichment analysis is conducted across three GO categories, including biological process (GO:BP), molecular function (GO:MF), and cellular component (GO:CC), based on the GO_Biological_Process_2021, GO_Molecular_Function_2021, and GO_Cellular_Component_2021 gene sets. The organism is set to human, and a significance threshold of p < 0.05 is applied to identify enriched terms. The results are saved for downstream analysis and visualization. This analysis enables systematic characterization of functional themes and biological pathways associated with spatially variable genes, providing insights into the molecular basis of spatial heterogeneity.

In summary, these analyses provided functional evidence for evaluating the biological relevance of fine-grained spatial domains identified by TRACER.

2 **Supplementary File 2: Details of the** **parameter settings**

TRACER is implemented based on *PyTorch* (v2.2.2, CUDA 11.8) with Python 3.10.16, and all experiments are conducted on a server equipped with an NVIDIA Tesla A100 GPU. The core architecture of TRACER is a single-layer graph convolutional network (GCN) autoencoder, with an embedding dimension of 128. According to systematic benchmarking, for 10x Visium datasets, the top 3,000 highly variable genes are selected, and the number of neighbors is set to 3. For sequencing platforms with a limited number of genes, such as osmFISH and STARmap, all genes were included in the analysis, and a smaller embedding dimension together with a larger neighborhood size was used according to fixed platform-level rules to avoid over-parameterization and improve graph connectivity. The recommended values for the loss function weights *α*, *β*, and *γ* are 10, 0.2, and 0.5, respectively. The model is optimized using the Adam optimizer with a learning rate of 0.001 and a weight decay of 0. Training is performed for 500 epochs. All baseline methods were run using the author-recommended settings from their original publications and official implementations, or the default settings in the released code when available. No additional dataset-specific hyperparameter tuning was performed for baseline methods, and all methods were evaluated using the same preprocessing and evaluation pipeline.

3 **Supplementary File 3: Definition of benchmarking evaluation metrics**

To evaluate the performance of spatial domain identification, we adopt the Adjusted Rand Index (ARI) and Normalized Mutual Information (NMI) as evaluation metrics.

The Adjusted Rand Index (ARI) is a chance-corrected version of the Rand Index (RI), which measures the agreement between predicted clustering results and ground-truth labels. Its value ranges from $\left[ -1,1 \right]$, where values close to 1 indicate high consistency, values around 0 correspond to random assignments, and negative values indicate systematic disagreement. ARI is defined as：

$ARI\left( U,V \right)=\frac{RI\left( U,V \right)\mathbb{-E}\left[ RI\left( U,V \right) \right]}{max\left( RI\left( U,V \right)\mathbb{-E}\left[ RI\left( U,V \right) \right] \right)}$	(1)

where $U$and $V$denote the ground-truth labels and predicted clustering results, respectively, and $\mathbb{E}[\cdot]$represents the expected value. The Rand Index (RI) is defined as:

$RI\left( U,V \right)=\frac{TP+TN}{TP+TN+FP+FN}$	(2)

where $TP$, $TN$, $FP$, and $FN$represent the numbers of true positive, true negative, false positive, and false negative pairs, respectively.

Normalized Mutual Information (NMI) measures the shared information between predicted labels and ground-truth labels, with normalization to account for differences in cluster sizes. Its value ranges from$\left[ 0,1 \right]$, where higher values indicate better agreement. NMI is defined as:

$NMI\left( U,V \right)=\frac{MI\left( U,V \right)}{\sqrt{H\left( U \right)H\left( V \right)}}$	(3)

where $MI(\cdot)$denotes mutual information and $H(\cdot)$denotes entropy.

4 **Supplementary File 4: Dataset descriptions and data access links**

**Table S1. Description of all ST datasets used in this study**

| **Platform** | **Tissue** | **Section** | **Spots** | **Genes** | **URLs** |
| --- | --- | --- | --- | --- | --- |
| 10x Visium | DLPFC | 151507 | 4226 | 33538 | http://research.libd.org/spatialLIBD/ |
|  |  | 151508 | 4384 |  |  |
|  |  | 151509 | 4789 |  |  |
|  |  | 151510 | 4634 |  |  |
|  |  | 151669 | 3661 |  |  |
|  |  | 151670 | 3498 |  |  |
|  |  | 151671 | 4110 |  |  |
|  |  | 151672 | 4015 |  |  |
|  |  | 151673 | 3639 |  |  |
|  |  | 151674 | 3673 |  |  |
|  |  | 151675 | 3592 |  |  |
|  |  | 151676 | 3460 |  |  |
|  | Mouse anterior brain | Not applicable | 2695 | 32285 | https://doi.org/10.5281/zenodo.10968451. |
| STARmap | Mouse primary visual cortex | Not applicable | 1207 | 1020 | https://sysbio.gzzoc.com/Mouse-Brain-Aging/ |
| osmFISH | Mouse somatosensory cortex | Not applicable | 4,839 | 33 | https://github.com/JinmiaoChenLab/SEDR_analyses/tree/master/data |
|  |  | 1 | 1525 | 79 |  |
| BARISTAseq | Mouse visual cortex | 2 | 2042 | 79 | http://sdmbench.drai.cn/ |
|  |  | 3 | 1690 | 79 |  |
| 10 x Visium | Human Breast Cancer | Not applicable | 3798 | 36601 | https://support.10xgenomics.com/spatial-gene-expression/datasets |

5 **Supplementary File 5: The Supplementary figures.**

**Figure S1. Spatial domain identification performance on slices #151507~#151510.**

**Figure S2. Spatial domain identification performance on slices #151669~#151672.**

**Figure S3. Spatial domain identification performance on slices#151673~#151674**

**Figure S4. Spatial domain identification results of TRACER and benchmark methods on two additional slices of the BARISTA dataset.**

**Figure S5. The proportion of spots for each spatial domain on the osmFISH dataset.**

**Figure S6. UMAP results of TRACER and SEDR on rare spatial domain datasets.**

**Figure S7. Cross-resolution stability analysis of fine-grained spatial subdomains within the Healthy_1 region.** The clustering number $K$was varied from 15 to 45 using the same TRACER-learned low-dimensional representation, and clustree was applied to visualize cluster inheritance, splitting, and reorganization across adjacent $K$values. At $K=20$, the Healthy_1-associated region was divided into domain 13 and domain 19. With increasing $K$, domain 13 was stably inherited and mainly corresponded to domain 17 at $K=40$, whereas domain 19 gradually split into domains 22, 30, and 4. These branches remained stable when $K$was further increased from 40 to 45. The results suggest that the fine-grained subdomains observed at $K=40$are not resolution-specific artifacts, but stable structures continuously traceable across clustering resolutions.

**Figure S8. The proportion of spots for each spatial domain in the BRCA dataset under the clustering number of 40.**

**Figure S9. Enrichment analysis results for spatial domains 4, 17, 22, and 30 versus all other domains.**

**Figure S10. The enrichment analysis results of spatial domain 17 versus all other domains.**

**Figure S11. The enrichment analysis results of spatial domain 4 versus other domains.**

**Figure S12. Enrichment analysis results for spatial domain 4 versus spatial domain 17.**

**Figure S13. Enrichment analysis results for spatial domain 22 versus all other domains.**

**Figure S14. Enrichment analysis results for spatial domain 22 versus spatial domain 17.**

**Figure S15. Enrichment analysis results for spatial domain 30 versus all other domains.**

**Figure S16. Enrichment analysis results for spatial domain 30 versus spatial domain 17.**

**Figure S17. Ablation experimental results on rare spatial domain datasets.**

**Figure S18. The runtime efficiency and peak GPU memory consumption of TRACER and benchmark methods on the DLPFC dataset.**

6 **Supplementary File 6: Details of the analysis results of small spatial domains on** **human breast cancer tissue**

In this experiment, we analyzed a human breast cancer spatial transcriptomics dataset, in which the expression of 36,601 genes was detected. Based on H&E-stained images and breast cancer-related marker genes, pathologists manually annotated the tissue into 20 spatial regions, including invasive ductal carcinoma (IDC), ductal carcinoma in situ/lobular carcinoma in situ (DCIS/LCIS), normal tissue, and tumor border regions (Figs. 5A and 5B). On this dataset, we compared TRACER with multiple benchmark methods. The results showed that TRACER achieved the best performance in spatial domain identification, with an ARI of 0.61 and an NMI of 0.71, both of which outperformed the competing methods (Figs. 5C and 5D).

Inspection of the TRACER clustering results suggested that the upper-right region annotated as Healthy_1 may contain additional internal spatial heterogeneity. Because fine-grained clustering can be sensitive to the choice of cluster number K, we performed a cross-resolution stability analysis to evaluate whether the observed substructure was stable across different clustering resolutions. Specifically, we varied K from 15 to 45 while keeping the learned TRACER embedding fixed, and used clustree to visualize the inheritance, splitting, and reorganization patterns of clusters across adjacent K values(Supplementary Fig. S7).

The clustree analysis showed that the Healthy_1-associated clusters followed continuous inheritance trajectories as K increased, without obvious large-scale cross-mixing or frequent reorganization. In particular, the subdomains observed at K=40 could be traced from lower-resolution clustering results and remained largely stable when K was further increased. These results suggest that the fine-grained subdivisions within the Healthy_1 region are not solely driven by a single arbitrary choice of K, but instead show cross-resolution stability.

Based on this stability analysis, we selected the K=40 as the result for exploratory downstream characterization. At K=40, the Healthy_1-associated region was subdivided into four spatial domains: 4, 17, 22, and 30 (Fig. 5E, F). Among them, domains 4, 22, and 30 accounted for only 3.7%, 0.6%, and 1.4% of all spots, respectively, and were therefore considered small spatial domains in this analysis (Supplementary Fig. S8). Together, these results provide evidence of stable fine-grained spatial heterogeneity within the Healthy_1 region, although these subdomains should be interpreted as exploratory findings rather than definitive biological annotations in the absence of independent orthogonal validation.

We next examined whether these small spatial domains showed biologically interpretable molecular differences rather than being merely arbitrary over-clustering results. Spearman correlation analysis showed that domains 4, 22, and 30 were more similar to one another than to domain 17 (Fig. 5G). To further characterize the biological functions of these spatial domains, we performed pairwise differential expression analysis among domains 4, 17, 22, and 30. For each pairwise comparison, differentially expressed genes were separated according to the direction of regulation. Specifically, genes up-regulated in one domain relative to the compared domain were used for pathway enrichment analysis of that domain, whereas genes up-regulated in the other domain were analyzed separately. Therefore, pathway enrichment was conducted using domain-specific up-regulated genes rather than pooled differentially expressed genes.

Based on these domain-specific up-regulated genes, enriched terms for domains 4, 17, 22, and 30 were mainly associated with extracellular matrix organization, structural maintenance (Brassart-Pasco *et al.* 2020) and microenvironment remodeling (Fig. 5H; Supplementary Fig. S9). Domain 17 showed a distinct functional profile based on its up-regulated genes, including protein metabolic modification, cell activation, motility, proliferation, and inflammatory signaling (Supplementary Fig. S10).

Based on these observations, we next focused on domains 4, 22, and 30. For each domain, two comparative analyses were performed: one comparing the domain with all remaining spatial domains, and the other comparing it specifically with domain 17. Based on these comparisons, GO Biological Process (BP), GO Cellular Component (CC), and GO Molecular Function (MF) enrichment analyses were conducted to systematically characterize the molecular features of these small spatial domains and their functional differences from those of domain 17.

Domain 4 was primarily located in the upper internal region of domain 17, forming a relatively compact, spatially clustered structure embedded within the main body of domain 17. Enrichment analysis showed that in the comparison between domain 4 and all other spatial domains, the differentially expressed genes were mainly enriched in processes related to extracellular matrix organization, extracellular structure maintenance, and supramolecular fiber organization, together with functions associated with secretory output, cell adhesion, and growth factor-related signaling, indicating pronounced features of matrix organization and structural maintenance (Fig. 5I and Supplementary Fig. S11). In the direct comparison between domain 4 and domain 17, domain 4 retained these extracellular matrix- and structure-related functional characteristics, while also showing more prominent signals for protein metabolism, translation, and membrane protein targeting and transport (Fig. 5J and Supplementary Fig. S12). Taken together, the two analyses indicate that matrix organization and structural maintenance represent stable shared features of domain 4 across different comparative settings, reflecting its consistent core functional identity. In contrast, compared with the broader structural enrichment observed when domain 4 was compared with all other domains, its distinction from domain 17 was more specifically characterized by enhanced protein metabolism and membrane protein transport activity. Considering its spatial localization within domain 17, domain 4 may represent an internal functional subregion characterized by structural homeostasis, accompanied by locally enhanced signaling regulation and secretory activity.

Domain 22 was also located at the periphery of the area covered by domain 17, but compared with domain 4, it was positioned closer to the right boundary and showed a more restricted local distribution. Enrichment analysis showed that in the comparison between domain 22 and all other spatial domains, the differentially expressed genes were enriched not only in extracellular matrix organization and structural maintenance, but also more prominently in functions related to protein synthesis, ribosomal activity, membrane transport, and secretory output, suggesting that this domain exhibits not only certain matrix-related characteristics but also a relatively active state of biosynthesis and material export Supplementary Fig. S13). In the direct comparison between domain 22 and domain 17, the enrichment of translation, membrane transport, and secretory processing remained evident, indicating that these functions represent stable molecular features distinguishing domain 22 from the surrounding main region (Supplementary Fig. S14). Overall, the two analyses suggest that protein synthesis, membrane transport, and secretory output activity are the most consistent core features of domain 22. The main difference between the two comparisons is that when domain 22 was compared with all other domains, it also showed a certain degree of enrichment in matrix- and structure-related functions, whereas in comparison with domain 17, these structural signals were relatively attenuated and the differences were more concentrated in enhanced translation and secretory processing. Given its localized distribution at the edge of the main region, domain 22 may represent a peripheral microregion within Healthy_1 characterized by relatively elevated metabolic and secretory activity.

Domain 30 was mainly distributed along the lower-right boundary of domain 17. Although spatially adjacent to domain 22, it formed a relatively independent small cluster. Enrichment analysis showed that in the comparison between domain 30 and all other spatial domains, its differentially expressed genes were mainly enriched in functions related to collagen-associated matrix remodeling, collagen fibril organization, local migration regulation, angiogenesis regulation, and peptidase activity modulation, together with enrichment of secretory processing and lysosome-related cellular components, suggesting a strong feature of dynamic microenvironment remodeling. The results are shown in Supplementary Fig. S15. In the direct comparison between domain 30 and domain 17, these features—including collagen remodeling, peptidase regulation, and secretory processing—remained prominent, indicating that they constitute the major molecular basis distinguishing domain 30 from the main region (Supplementary Fig. S16). Taken together, the two analyses indicate that matrix remodeling and microenvironmental regulation are stable functional features of domain 30. The difference between the two comparisons is that, compared with all other domains, domain 30 showed broader enrichment in migration-related regulation, angiogenesis-related regulation, and lysosome-associated cellular components, whereas, compared with domain 17, its distinguishing features were more specifically reflected in enhanced fibrotic matrix remodeling and peptidase-related regulatory activity. Given its peripheral localization adjacent to domain 22, domain 30 may represent a boundary microregion within Healthy_1 with a stronger tendency toward local tissue remodeling and dynamic microenvironmental regulation.

In summary, although domains 4, 22, and 30 were all located within the original Healthy_1 region and were spatially adjacent to, or partially embedded within, domain 17, they were not functionally identical. All three domains shared common features centered on extracellular matrix organization and structural maintenance, suggesting a certain degree of common underlying molecular identity. However, relative to domain 17, which constitutes the main body of the region, each domain exhibited a distinct functional bias. Specifically, domain 4, located in the upper internal region, was more associated with structural homeostasis and related signaling regulation; domain 22, located at the right boundary, was characterized more prominently by protein synthesis and secretory output activity; and domain 30, located at the lower-right boundary adjacent to domain 22, was more strongly associated with collagen remodeling and peptidase-related regulation. These results indicate that the region originally annotated at the macroscopic level as Healthy_1 comprises multiple small spatial domains with distinct spatial localizations and functional specializations, further demonstrating our method's ability to resolve local spatial heterogeneity and uncover fine-grained functional stratification in complex tissue contexts.

7 **Supplementary File 7: Parameter analysis of TRACER**

For the loss weights in TRACER, we performed a detailed parameter sensitivity analysis. Specifically, we tested $\alpha\in\left\{ 1 , 5 , 10 \right\}$, while $\beta$and $\gamma$were each selected from $\left\{ 0.1 , 0.2 , 0.3 , \ldots, 1.0 \right\}$. This resulted in a total of 300 parameter combinations. Because including all results in the main text or supplementary files would be excessively lengthy, we uploaded the complete sensitivity analysis results to the GitHub repository (<https://github.com/luxin-heart/TRACER>) to ensure transparency and reproducibility.

In addition, we evaluated the effect of the hyperparameter $\lambda$on the DLPFC dataset. As shown in Table S2, TRACER achieved the best overall performance when $\lambda=1$, with a mean ARI of 0.65 and a median ARI of 0.66.

**Table S2. Performance of TRACER on the DLPFC dataset under different λ**

| **λ** | **Mean ARI** | **Median ARI** |
| --- | --- | --- |
| 0.1 | 0.61 | 0.61 |
| 1 | 0.65 | 0.66 |
| 10 | 0.57 | 0.56 |

8 **Supplementary File 8: Ablation experiments of TRACER**

This supplementary file reports ablation experiments for TRACER, including the contribution of the instance-level and cluster-level contrastive learning modules, as well as the effect of the post-processing refinement strategy. We further compare TRACER with benchmark methods on the DLPFC dataset with and without post-processing.

We first evaluated the contribution of the instance-level and cluster-level contrastive learning modules on two rare spatial domain subsets (Here, Dataset 1 denotes the non-cortical rare-domain subset, and Dataset 2 denotes the cortical transition-layer subset). The results show that removing either module led to performance degradation, and removing both modules resulted in the largest decrease in both ARI and NMI, indicating that the two contrastive learning modules contribute jointly to the performance of TRACER.

**Table S3. Ablation experiments of TRACER on rare spatial domain subset 1.**

| **Ablation Module** | **ARI** | **NMI** |
| --- | --- | --- |
| w/o Instance | 0.57 | 0.41 |
| w/o Cluster | 0.58 | 0.42 |
| w/o Both | 0.46 | 0.32 |

**Table S4. Ablation experiments of TRACER on rare spatial domain subset 2.**

| **Ablation Module** | **ARI** | **NMI** |
| --- | --- | --- |
| w/o Instance | 0.64 | 0.55 |
| w/o Cluster | 0.67 | 0.54 |
| w/o Both | 0.54 | 0.42 |

We next evaluated TRACER and benchmark methods on the DLPFC dataset using post-processing refinement. As shown in Table S5, TRACER achieved the highest mean ARI and median ARI among all compared methods.

**Table S5. Performance of TRACER and benchmark methods on the DLPFC dataset with post-processing refinement.**

| **Method** | **Mean ARI** | **Median ARI** |
| --- | --- | --- |
| TRACER | 0.65 | 0.66 |
| GraphST | 0.55 | 0.58 |
| SEDR | 0.51 | 0.54 |
| STAGATE | 0.53 | 0.53 |
| STMIGCL | 0.47 | 0.49 |
| stDCL | 0.53 | 0.52 |
| DenoiseST | 0.53 | 0.49 |

To further assess whether the performance improvement of TRACER is solely due to post-processing, we compared all methods on the DLPFC dataset without using post-processing refinement. As shown in Table S6, TRACER still achieved competitive performance and obtained the highest mean ARI, although the performance gap was smaller than that observed with post-processing.

**Table S6. Performance of TRACER and benchmark methods on the DLPFC dataset without post-processing refinement.**

| **Method** | **Mean ARI** | **Median ARI** |
| --- | --- | --- |
| TRACER | 0.54 | 0.57 |
| GraphST | 0.5 | 0.5 |
| SEDR | 0.53 | 0.55 |
| STAGATE | 0.46 | 0.46 |
| STMIGCL | 0.41 | 0.41 |
| stDCL | 0.52 | 0.53 |
| DenoiseST | 0.52 | 0.51 |

Finally, we evaluated the effect of post-processing refinement on the two rare spatial domain subsets using TRACER. As shown in Table S7, post-processing refinement improved the ARI on both subsets, suggesting that it can further enhance spatial domain assignment by incorporating local spatial consistency.

**Table S7. Ablation experiments of post-processing refinement on rare spatial domain subsets using TRACER.**

| **Dataset** | **Refine** | **Without_refine** |
| --- | --- | --- |
| Dataset 1 | 0.63 | 0.58 |
| Dataset 2 | 0.73 | 0.64 |

9 **Supplementary File 9: Architecture and Augmentation Sensitivity Analysis**

This supplementary file reports additional sensitivity analyses of TRACER with respect to model architecture and graph augmentation strategy. Specifically, we evaluated the effects of GCN depth, GNN backbone choice, and augmentation strategy on the DLPFC dataset. The results show that the default TRACER configuration, namely a one-layer GCN encoder with feature-vector shuffling, achieved the best overall performance.

We first examined the influence of GCN depth on TRACER performance. As shown in Table S8, increasing the number of GCN layers led to a clear decrease in performance. In particular, three-layer GCN encoders performed substantially worse, even when residual connections were added. These results support the use of a one-layer GCN encoder in TRACER, possibly because deeper GCNs introduce excessive spatial smoothing and obscure fine-grained spatial domain boundaries.

**Table S8. Performance of TRACER on the DLPFC dataset with different GCN depths.**

| **GCN layers** | **Mean ARI** | **Median ARI** |
| --- | --- | --- |
| One layer | 0.65 | 0.66 |
| Two layers | 0.49 | 0.46 |
| Three layers without residual connection | 0.27 | 0.29 |
| Three layers with residual connection | 0.34 | 0.34 |

We next evaluated whether TRACER depends on a specific graph neural network backbone. As shown in Table S9, the GCN-based encoder achieved the highest mean and median ARI among the tested backbones, while GAT and GraphSAGE also produced reasonable performance. These results suggest that TRACER can be implemented with different GNN backbones, while the GCN encoder provides the most stable and effective performance in our experiments.

**Table S9. Performance of TRACER on the DLPFC dataset with different GNN backbones.**

| **GNN backbone** | **Mean ARI** | **Median ARI** |
| --- | --- | --- |
| GCN | 0.65 | 0.66 |
| GAT | 0.58 | 0.61 |
| GraphSAGE | 0.53 | 0.56 |

Finally, we compared different graph augmentation strategies. As shown in Table S10, feature-vector shuffling achieved the best performance, whereas edge dropping, feature masking, and their combination led to lower ARI values. For edge dropping and feature masking, we followed the masking and edge-drop probabilities used in STAIG, so that these augmentation baselines were evaluated under established parameter settings. These results indicate that the augmentation strategy has a substantial influence on representation learning and downstream spatial domain identification. The superior performance of feature-vector shuffling suggests that it provides an effective perturbation for contrastive learning while preserving the spatial graph structure.

**Table S10. Performance of TRACER on the DLPFC dataset with different graph augmentation strategies.**

| **Augmentation strategy** | **Mean ARI** | **Median ARI** |
| --- | --- | --- |
| Feature-vector shuffling | 0.65 | 0.66 |
| Edge dropping | 0.54 | 0.57 |
| Feature masking | 0.5 | 0.53 |
| Edge dropping + feature masking | 0.53 | 0.54 |

10 **Supplementary File 10: Random Seed Robustness Analysis**

This supplementary file evaluates the robustness of TRACER to random initialization. We tested TRACER using multiple random seeds and compared its performance on the DLPFC dataset. As shown in Table S11, TRACER showed only limited variation across different random seeds, with mean ARI values ranging from 0.60 to 0.65 and median ARI values ranging from 0.63 to 0.66. These results indicate that TRACER maintains relatively stable performance under different random initializations.

**Table S11. Performance of TRACER on the DLPFC dataset under different random seeds.**

| **Ramdom Seed** | **Mean ARI** | **Median ARI** |
| --- | --- | --- |
| 0 | 0.62 | 0.63 |
| 1 | 0.62 | 0.64 |
| 3 | 0.63 | 0.64 |
| 41 | 0.6 | 0.63 |
| 42 | 0.64 | 0.65 |
| 2025 | 0.65 | 0.66 |

11 **Supplementary File 11: Computational Efficiency and Scalability Analysis**

This supplementary file reports the computational efficiency and scalability of TRACER. We first compared the runtime and peak GPU memory usage of TRACER with benchmark methods on the DLPFC dataset under the same computational environment. As shown in Table S11, TRACER required 726 seconds with a peak GPU memory usage of 1.15 GB. Although TRACER was not the fastest method in terms of runtime, it achieved the lowest GPU memory usage among the compared methods. This suggests that TRACER is memory-efficient on the DLPFC dataset, which may be advantageous for analyses on hardware with limited GPU memory.

**Table S12. Runtime and peak GPU memory usage of TRACER and benchmark methods on the DLPFC dataset.**

| **Method** | **Runtime (s)** | **Peak GPU memory (GB)** |
| --- | --- | --- |
| TRACER | 726 | 1.15 |
| GraphST | 323 | 9.65 |
| SEDR | 432 | 5.22 |
| STAGATE | 583 | 4.28 |
| stDCL | 593 | 8.53 |
| DenoiseST | 335 | 4.32 |

We further evaluated the computational scalability of TRACER with respect to the number of spatial spots. Specifically, we measured the runtime and peak GPU memory usage of TRACER on datasets with different numbers of spots. As shown in Table S13, both runtime and peak GPU memory usage increased as the number of spatial spots increased. TRACER completed the analysis of 43,560 spots in 450.12 seconds, with a peak GPU memory usage of 65.48 GB. These results suggest that TRACER remains applicable to large-scale spatial transcriptomics datasets, although its computational cost increases with dataset size.

**Table S13. Runtime and peak GPU memory usage of TRACER under different numbers of spatial spots.**

| **Number of spots** | **Runtime (s)** | **Peak GPU memory (GB)** |
| --- | --- | --- |
| 1207 | 18.59 | 0.15 |
| 2695 | 31.41 | 0.52 |
| 4839 | 54.34 | 1.13 |
| 5926 | 66.72 | 1.33 |
| 8186 | 88.75 | 2.45 |
| 10890 | 116.50 | 4.24 |
| 21780 | 227.71 | 16.37 |
| 32670 | 338.92 | 38.16 |
| 43560 | 450.12 | 65.48 |
